# Characterization and evaluation of Nepalese rice landraces using agro-morphological traits

**DOI:** 10.1371/journal.pone.0348162

**Published:** 2026-08-03

**Authors:** Surakshya Ghimire, Rajan Lamsal, Gautam Thapa, Santosh Marahatta

**Affiliations:** 1 Department of Genetics and Plant Breeding, Agriculture and Forestry University, Chitwan, Nepal; 2 Department of Plant Pathology, Agriculture and Forestry University, Chitwan, Nepal; 3 Department of Agronomy, Agriculture and Forestry University, Chitwan, Nepal; Shahjalal University of Science and Technology, BANGLADESH

## Abstract

Rice (Oryza sativa L.) landraces represent an important reservoir of genetic diversity for crop improvement. To characterize agro-morphological variation, a total of 63 rice landraces were evaluated using qualitative and quantitative descriptors, with the improved variety Lalka Basmati included as a check. Among 29 qualitative traits assessed, basal leaf sheath color, leaf blade intensity of green color, auricle color, flag leaf blade attitude, and culm lodging resistance exhibited the highest variation, whereas ligule shape showed no variation across landraces. Highly significant differences (p < 0.001) were observed for key quantitative traits, including flag leaf length and width, penultimate leaf dimensions, ligule length, culm length and diameter, culm number per plant, panicle number and length, filled grains per panicle, total and productive tillers per square meter, thousand-grain weight, grain dimensions, straw yield, grain yield, and harvest index. Vhadasar (3397 kg ha^−1^) and Ratin (3396 kg ha^−1^) were identified as high-yielding landraces with superior harvest indices. High genotypic and phenotypic coefficients of variation coupled with high heritability and genetic advance as a percentage of mean were recorded for flag leaf traits, culm length, culm number per plant, and total tillers per square meter, indicating substantial scope for genetic improvement. Grain yield exhibited significant positive correlations with panicle number per plant, filled grains per panicle, culm number per plant, and grain length and width. Principal component analysis revealed that the first five components explained 73.40% of the total phenotypic variation. Cluster analysis using unweighted pair group method with arithmetic mean (UPGMA) grouped the landraces into eight distinct clusters, reflecting varying degrees of genetic relatedness. The observed level of agro-morphological diversity highlights the potential of these rice landraces for use in selective breeding and genetic improvement programs.

## Introduction

Rice (Oryza sativa L.; 2n = 24) is one of the world’s most important cereal crops, serving as the primary staple food for more than half of the global population. It belongs to the family Poaceae and subfamily Oryzoideae, classified into three main subspecies: indica, japonica, and javanica. Rice was first domesticated in tropical Asia around 5000 BC and subsequently spread to temperate regions worldwide [1]. Asia accounts for over 90% of global rice production and consumption, underscoring its critical role in food security [2]. Globally, rice is cultivated on approximately 165 million hectares, with a total paddy production of 776 million metric tons and an average yield of 4.7 t ha^−1^ [3]. Effective crop establishment and management practices, including direct seeding and integrated weed management, significantly influence rice growth and productivity [4], while disease resistance in landraces remains crucial for maintaining stable yields under field conditions [5]. Moreover, comprehensive phenotypic and molecular assessments of rice landraces have revealed wide genetic diversity with plant height ranging from 85 to 165 cm and expected heterozygosity of 0.42 to 0.78, underscoring the breadth of available variation for breeding [6].

In Nepal, rice is the most widely cultivated crop, covering 1.44 million hectares with an annual production of 5.72 million metric tons and an average productivity of 4.0 t ha^−1^ [7]. It contributes approximately 20% to the agricultural GDP and 7% to the national GDP, while supplying around 40% of the daily caloric intake for the Nepali population [8,9] with over 53% of households directly involved in rice cultivation [10], rice availability and affordability are directly linked to national food security and economic stability. However, despite recent improvements, Nepal’s rice productivity remains lower than neighboring countries such as China (7.1 t ha^−1^) and India (4.2 t ha^−1^), making yield enhancement a priority for improving farmer livelihoods and achieving national food self-sufficiency [11].

Nepal’s diverse agro-ecological zones—ranging from the Terai lowlands (67–900 m above sea level, masl) to the High Hills (1500–3050 masl)—combined with variation in climate, soils, and traditional farming practices, have resulted in a rich collection of rice landraces [12,13]. These landraces are genetically diverse, locally adapted, and represent a valuable source of traits for breeding and conservation [14,15]. Compared with modern improved varieties, landraces maintain higher genetic variation and provide unique opportunities to introduce novel alleles for yield improvement, stress tolerance, and grain quality into breeding programs [16,17]. Community seed banks across Nepal serve as critical repositories of this genetic diversity, preserving landraces that may otherwise be lost due to the widespread adoption of modern varieties [18]. Recent studies across South Asia have reinforced the importance of systematic landrace characterization; in Bangladesh, morpho-molecular evaluation of rice landraces revealed high genetic diversity suitable for breeding programs [19], while agro-morphological characterization of 47 Nepalese rice landraces at Rampur, Chitwan confirmed significant variation for yield-related traits and identified promising genotypes through UPGMA clustering [20]. Additionally, about 33% of landrace diversity in Nepal has been lost in recent decades due to replacement by modern high-yielding varieties, making conservation efforts increasingly urgent [6].

Although previous studies in Nepal have examined yield or individual traits of rice germplasm [21,22], and recent work has addressed crop management practices such as direct-seeded rice establishment [4] and disease resistance screening of landraces [5], systematic evaluations of landraces using both qualitative and quantitative agro-morphological descriptors remain limited. Furthermore, relationships among yield, component traits, and genetic diversity patterns have not been fully explored, leaving critical gaps in the identification of promising landraces for breeding and conservation programs. To address these gaps, the present study was conducted to: (i) characterize Nepalese rice landraces using comprehensive agro-morphological traits, (ii) estimate genetic variability, heritability, and genetic advance for yield-related traits, (iii) analyze correlations among yield and its component traits, and (iv) assess genetic diversity patterns through multivariate analyses. The overall goal is to identify high-yielding, well-adapted landraces that can serve as genetic resources for future rice improvement and conservation programs in Nepal.

## Materials and methods

### Selection of site

The field experiment was conducted at the Agriculture and Forestry University (AFU) research farm in Rampur, Chitwan, Nepal. The precise location of the experimental site was 27°37′N latitude, 84°25′E longitude, and at an altitude of 228 meters above sea level. The experimental site is located in the Inner Terai agro-ecological zone, characterized by alluvial silty loam soil (pH 6.0) with moderate fertility and good drainage properties. The region experiences a subtropical monsoon climate with distinct wet (June–September) and dry (October–May) seasons. The growing season (June–November 2018) experienced typical monsoon conditions with total rainfall of 1,847 mm, mean temperature of 26.8°C, and relative humidity ranging from 65% to 95%.

### Ethics statement

This study did not involve human participants, animals, or protected species, and no ethical approval or consent was required. The rice landraces were obtained, with permission, from community seed banks in Jhapa, Bara, Nawalparasi, and Dang districts, Nepal.

### Experimental design and data collection

The field trial was conducted under irrigated transplant conditions. Twenty-five-day-old seedlings were transplanted into the field in an alpha-lattice design with three replications. Each replication contained eight blocks, and each block had eight genotypes with a plot size of (2 m × 1 m) 2 m^2^. The crop geometry was 20 cm × 20 cm between plants and rows. One meter space was maintained between replications and 0.5 m between each block. Two to three seedlings were transplanted per hill in five rows per plot. This planting density (two to three seedlings per hill at 20 × 20 cm spacing) was kept uniform across all genotypes and plots; therefore, differences in tiller number among genotypes reflected genotypic variation rather than differences in planting. Total and productive tillers were recorded per square meter, and filled grains per panicle, so that these traits represent stand- and panicle-level measures that do not depend on the number of seedlings established per hill.

Well-decomposed farmyard manure (FYM) was uniformly incorporated at 6 t ha^−1^ during final land preparation. No inorganic fertilizers were applied to maintain uniform organic cultivation conditions. The first and second weeding were carried out manually at 30 and 60 days after transplanting, respectively. Irrigation was performed regularly based on rainfall patterns and crop physiological requirements, maintaining 2–5 cm standing water during active tillering and reproductive phases. Additional agronomic practices, including gap filling at 7 days after transplanting and bund maintenance, were carried out uniformly across all plots, and no chemical pesticides or fungicides were applied.

A total of 29 qualitative characteristics and 21 quantitative traits were recorded to assess variability, following the standardized rice descriptors developed by Bioversity International, International Rice Research Institute (IRRI), and West Africa Rice Development Association (WARDA) [23]. Qualitative traits were scored by visual assessment against the defined descriptor states at the appropriate growth stages (late vegetative, anthesis, or near maturity, depending on the trait). Quantitative traits were measured on five randomly selected representative plants per plot using a measuring scale for linear leaf, culm, and panicle dimensions (cm), a vernier caliper and micrometer screw gauge for culm and grain dimensions (mm), and a precision balance for thousand-grain weight after drying to 13% moisture content. Grain and straw yields were recorded on a whole-plot basis; grain yield was adjusted to 13% moisture content using the standard moisture-correction formula and expressed in kg ha^−1^. Harvest index was calculated as the ratio of grain yield to biological yield (grain yield + straw yield), expressed as a percentage. The complete list of all traits, with their descriptor states or units, growth stage of recording, and method of observation, is provided in S1 Table and S2 Table.

### List of rice genotypes

A diverse collection of 64 rice genotypes comprising 63 traditional landraces and one released check variety (‘Lalka Basmati’) was evaluated. The landraces were collected from community seed banks across four major rice-growing districts of Nepal: Jhapa (n = 19), Bara (n = 32), Nawalparasi (n = 6), and Dang (n = 6), representing distinct agro-ecological zones and farmer selection practices (Table 1).

**Table 1:** List of 64 rice genotypes evaluated in the study with their source of collection.

SN	Genotype	Source	SN	Genotype	Source
1	Chhayang	Nawalparasi	33	Seto Dalle	Bara
2	Meethai	Nawalparasi	34	Siladhar	Bara
3	Rambela-An	Nawalparasi	35	Sthaniya Sadhi	Bara
4	Rato Anadi-Na	Nawalparasi	36	Ujarka Jehariya	Bara
5	Rato Basmati	Nawalparasi	37	Velasaro	Bara
6	Thapachini	Nawalparasi	38	Vhadasar	Bara
7	Dunminiya Seto	Bara	39	Lalka Basmati*	Check
8	Kalo Tulasi	Bara	40	Laalchand	Dang
9	Kanakirawi	Bara	41	Ratanpuri	Dang
10	Kanhar	Bara	42	Saauthyaari	Dang
11	Karijker	Bara	43	Simtharo	Dang
12	Karma	Bara	44	Thapachini	Dang
13	Kesarbachi	Bara	45	Tilaki	Dang
14	Khedra	Bara	46	Katar Khera	Jhapa
15	Kusumkali	Bara	47	Komal Dhan	Jhapa
16	Lalrenjer	Bara	48	Lalbachhi	Jhapa
17	Madhumala	Bara	49	Marshi Dhan	Jhapa
18	Malathe Ate	Bara	50	Najir	Jhapa
19	Malbhog	Bara	51	Pulingtar	Jhapa
20	Nakhisaro	Bara	52	Rambela-J	Jhapa
21	Pakha	Bara	53	Ranga Dhan	Jhapa
22	Pakhar	Bara	54	Ras Dhan	Jhapa
23	Parewapakh	Bara	55	Rato Anadi-J	Jhapa
24	Rajala	Bara	56	Rato Bachhi	Jhapa
25	Ramuni	Bara	57	Rato Sthaniya	Jhapa
26	Rango	Bara	58	Rudrakshya	Jhapa
27	Ratagola-1	Bara	59	Sadharan Kalo Nuniya	Jhapa
28	Ratagola-2	Bara	60	Samalchauri	Jhapa
29	Ratin	Bara	61	Sete Dhan	Jhapa
30	Rato Andi	Bara	62	Sikichan	Jhapa
31	Rato Tude Seto Basmati	Bara	63	Thulo Mansara	Jhapa
32	Sakhar	Bara	64	Tilki	Jhapa

*Check variety (released).

### Data analysis

MS Excel 2019 was used for data processing and data entry. The frequency distribution of the qualitative traits was calculated using SPSS version 25.0. For quantitative characters, R Studio and MINITAB 19.2 were used to analyze basic statistics, analysis of variance, pearson’s correlation, principal component analysis, and UPGMA cluster analysis based on Euclidean distance.

Variance components were estimated using restricted maximum likelihood (REML) to calculate genotypic (GCV) and phenotypic (PCV) coefficients of variation, broad-sense heritability (H^2^b), and genetic advance (GA). Classification criteria followed Johnson et al. [24]: low (<10%), moderate (10–20%), and high (>20%) for GCV, PCV, and GA as percentage of mean; low (<30%), moderate (30–60%), and high (>60%) for heritability.

## Results

### Variation in qualitative characters

**Fig 1:**
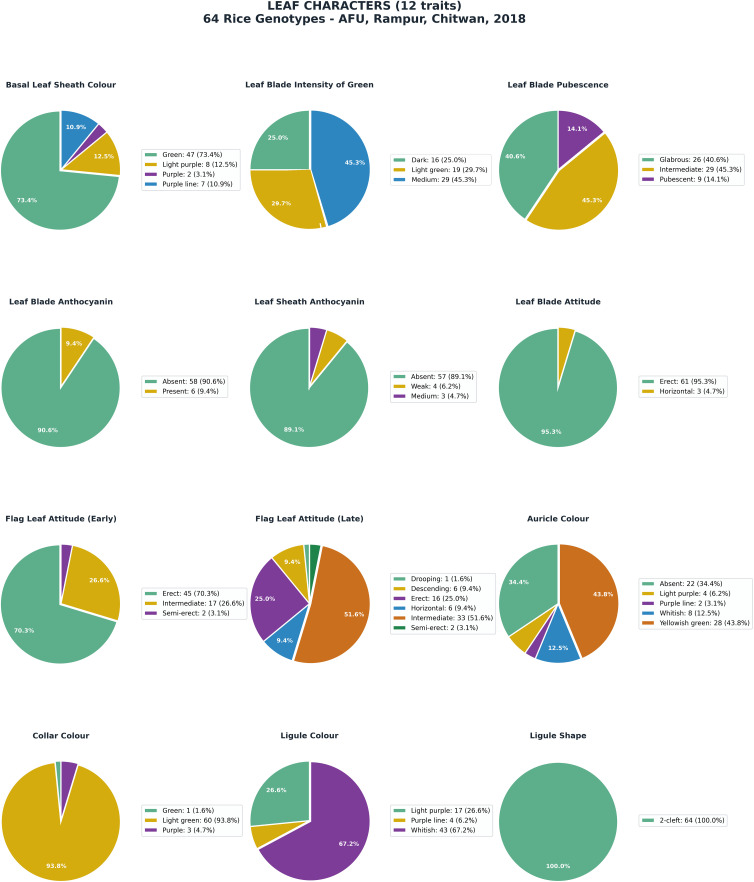
Leaf characters of 64 Nepalese rice landraces.

**Fig 2:**
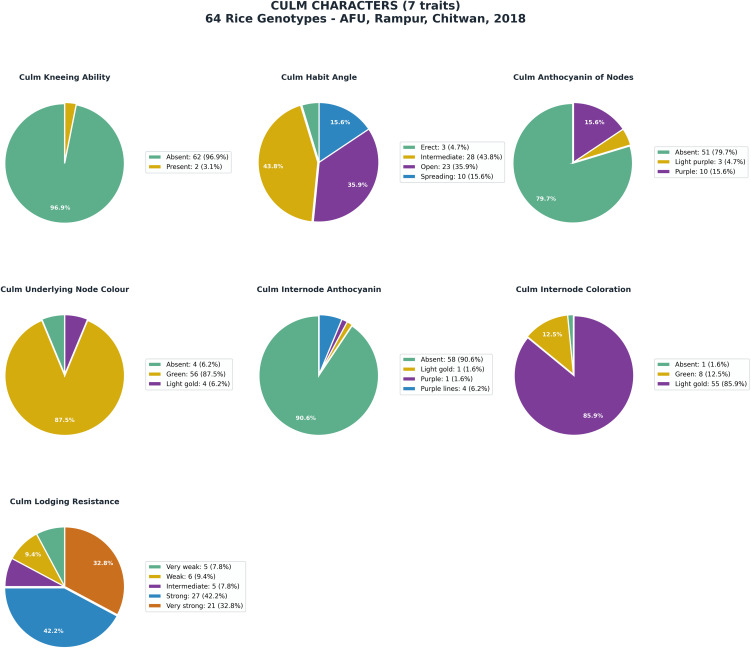
Culm characters of 64 Nepalese rice landraces.

**Fig 3:**
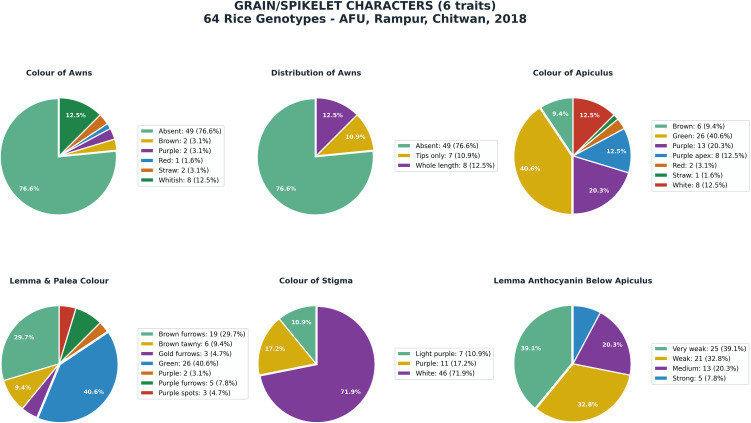
Grain characters of 64 Nepalese rice landraces.

**Fig 4:**
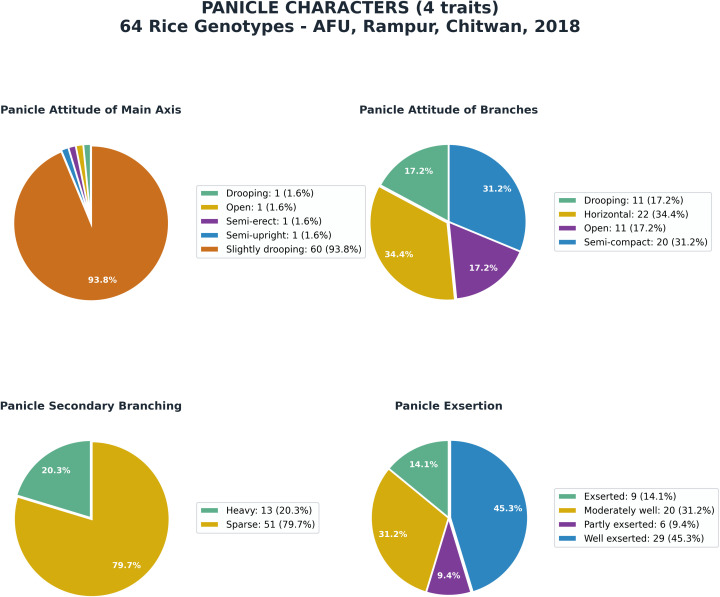
Panicle characters of 64 Nepalese rice landraces.

Significant polymorphism was observed for 28 of 29 qualitative traits evaluated. Only ligule shape remained monomorphic, with all 64 genotypes displaying 2-cleft ligules. The distribution of qualitative leaf, culm, grain, and panicle characters are presented in Figs 1–4.

***Leaf characters.*** The leaf characteristics are shown in Fig 1. Basal leaf sheath color showed that 47 (73.4%) of the 64 genotypes displayed green basal leaf sheath, whereas 8 (12.5%) had light purple, 7 (10.9%) had purple lines, and 2 (3.1%) had purple. Based on the intensity of the green color on the leaf blade, the genotypes were divided into three groups: 16 genotypes (25.0%) displayed a dark green color, 19 genotypes (29.68%) a light green color, and 29 genotypes (45.3%) a medium green color. Leaf blade anthocyanin coloration was absent in 58 genotypes (90.6%) and present in 6 genotypes (9.4%). Similarly, leaf sheath anthocyanin was absent in 57 (89.1%) of the genotypes, whereas it was present in 7 (10.9%). Leaf blade pubescence displayed great variety, with 26 (40.6%) genotypes glabrous, 29 (45.3%) possessing intermediate pubescence, and 9 (14.1%) pubescent. Leaf blade attitude was predominantly erect, observed in 61 genotypes (95.3%), with the remainder horizontal (3; 4.7%). Auricle color varied across the genotypes, with yellowish green auricles appearing in 28 (43.8%) and absent in 22 (34.4%), whitish in 8 (12.5%), light purple in 4 (6.2%) and purple-lined in 2 (3.1%). Light green collar color was observed in 60 genotypes (93.8%), purple in 3 (4.7%) and green in 1 (1.6%). There was no difference in the shape of the ligule, as all 64 genotypes (100%) had a 2-cleft shape, while ligule color was mostly whitish (43; 67.2%), light purple in 17 (26.6%) and purple-lined in 4 (6.2%). In the early reproductive stage, flag leaf attitude was erect in 45 genotypes (70.3%), intermediate in 17 (26.6%) and semi-erect in 2 (3.1%); at the late stage the intermediate type predominated (33; 51.6%), followed by erect (16; 25.0%), descending (6; 9.4%), horizontal (6; 9.4%), semi-erect (2; 3.1%) and drooping (1; 1.6%).

***Culm characters.*** The culm characteristics are shown in Fig 2. Culm kneeing ability was absent in 62 (96.9%) of the 64 genotypes, whereas it was present in only 2 (3.1%). Based on culm habit angle, the genotypes were divided into four groups: 28 genotypes (43.8%) showed an intermediate habit, 23 (35.9%) an open habit, 10 (15.6%) a spreading habit, and 3 (4.7%) an erect habit. Anthocyanin coloration of the nodes was absent in 51 genotypes (79.7%), whereas purple was found in 10 (15.6%) and light purple in 3 (4.7%); the underlying node color was predominantly green in 56 genotypes (87.5%). Culm internode anthocyanin was absent in 58 genotypes (90.6%), and the culm internode coloration was mostly light gold in 55 (85.9%). Culm lodging resistance displayed great variety, with 27 (42.2%) genotypes strong, 21 (32.8%) very strong, 6 (9.4%) weak, 5 (7.8%) very weak, and 5 (7.8%) intermediate, indicating good standability in the majority of the landraces.

***Grain characters.*** The grain and spikelet characteristics are shown in Fig 3. Awns were absent in 49 (76.6%) of the 64 genotypes, whereas among the awned types whitish awns appeared in 8 (12.5%), brown, purple, and straw in 2 each (3.1%), and red in 1 (1.6%). Correspondingly, awn distribution showed 49 genotypes (76.6%) awnless, 8 (12.5%) with awns over the whole length, and 7 (10.9%) with awns at the tips only. Apiculus color varied greatly across the genotypes, with green in 26 (40.6%), purple in 13 (20.3%), purple apex in 8 (12.5%), white in 8 (12.5%), brown in 6 (9.4%), red in 2 (3.1%), and straw in 1 (1.6%). Lemma and palea color also displayed wide variation, with green in 26 (40.6%), brown furrows in 19 (29.7%), brown tawny in 6 (9.4%), purple furrows in 5 (7.8%), purple spots in 3 (4.7%), gold furrows in 3 (4.7%), and purple in 2 (3.1%). Stigma color was predominantly white in 46 genotypes (71.9%), whereas purple appeared in 11 (17.2%) and light purple in 7 (10.9%). Based on the intensity of anthocyanin coloration below the apiculus, the genotypes were divided into very weak in 25 (39.1%), weak in 21 (32.8%), medium in 13 (20.3%), and strong in 5 (7.8%).

***Panicle characters.*** The panicle characteristics are shown in Fig 4. Panicle attitude of the main axis was predominantly slightly drooping, observed in 60 (93.8%) of the 64 genotypes, whereas drooping, open, semi-erect, and semi-upright types were each found in 1 genotype (1.6%). Panicle branch attitude displayed greater variety, with horizontal in 22 genotypes (34.4%), semi-compact in 20 (31.3%), drooping in 11 (17.2%), and open in 11 (17.2%). Secondary branching was sparse in 51 genotypes (79.7%) and heavy in 13 (20.3%). Based on panicle exsertion, the genotypes were divided into four groups: 29 (45.3%) were well exserted, 20 (31.3%) moderately well exserted, 9 (14.1%) exserted, and 6 (9.4%) partly exserted, indicating good panicle emergence in the majority of the landraces.

### Descriptive statistics of quantitative traits

The descriptive statistics for 21 quantitative traits of 64 rice genotypes are presented in Table 2. Wide range of variation was observed for all traits studied. Culm length ranged from 73.53 cm (Rato Sthaniya) to 138.20 cm (Dunminiya Seto) with a mean of 109.76 cm. Flag leaf length varied from 20.87 cm (Lalka Basmati) to 41.27 cm (Ratanpuri) with mean value of 29.55 cm. Panicle length ranged from 17.12 cm (Sthaniya Sadhi) to 26.83 cm (Ratagola-1) with a mean of 22.80 cm. Filled grains per panicle showed wide variation ranging from 11.60 (Dunminiya Seto) to 131.40 (Tilaki) with mean of 66.13. Thousand grain weight ranged from 18.73 g (Ras Dhan) to 30.85 g (Malathe ate) with mean of 24.60 g. Harvest index varied from 32.96% (Kesharbachi) to 78.14% (Thulo Mansara) with mean of 50.65%.

**Table 2:** Descriptive statistics of quantitative traits in 64 rice genotypes evaluated at AFU, Rampur, Chitwan, 2018.

Traits	Min	Max	Mean	CV (%)	LSD (0.05)
PLL (cm)	40.27	57.33	47.23	8.65	4.08
PLW (cm)	0.70	1.39	1.02	17.81	0.18
LLL (cm)	1.09	2.70	1.67	24.14	0.40
FLL (cm)	20.87	41.27	29.55	14.71	4.35
FLW (cm)	0.78	1.67	1.20	14.05	0.17
CL (cm)	73.53	138.20	109.76	8.42	9.24
CD (mm)	4.66	8.35	6.18	14.39	0.89
CNPP	1.71	19.00	10.25	27.87	2.86
PNPP	2.24	17.46	9.08	29.01	2.63
PL (cm)	17.12	26.83	22.80	6.53	1.49
FGPP	11.60	131.40	66.13	40.44	26.75
SSPP	9.00	89.00	34.31	42.55	14.60
SP (%)	7.42	80.15	35.71	38.48	13.74
GL (mm)	5.74	9.83	7.82	3.23	0.25
GW (mm)	1.39	1.94	1.66	5.17	0.09
TGW (g)	18.73	30.85	24.60	31.20	7.67
TTPM	127.00	252.00	189.47	18.74	35.51
PTPM	79.00	199.00	139.24	24.93	34.72
SY (kg/ha)	2665.00	6522.67	4341.52	3.26	141.52
GY (kg/ha)	433.00	3397.00	1847.32	46.75	863.54
HI (%)	32.96	78.14	50.65	26.57	13.46

PLL = Penultimate leaf length; PLW = Penultimate leaf width; LLL = Ligule length; FLL = Flag leaf length; FLW = Flag leaf width; CL = Culm length; CD = Culm diameter; CNPP = Culm number per plant; PNPP = Panicle number per plant; PL = Panicle length; FGPP = Filled grain per panicle; SSPP = Sterile spikelets per panicle; SP = Sterility percentage; GL = Grain length; GW = Grain width; TGW = Thousand grain weight; TTPM = Total tiller per m^2^; PTPM = Productive tiller per m^2^; SY = Straw yield; GY = Grain yield; HI = Harvest index; CV = Coefficient of variation; LSD = Least significant difference at 5% level.

The coefficient of variation (CV%) was highest for grain yield (46.75%) followed by sterile spikelets per panicle (42.55%), filled grains per panicle (40.44%), and sterility percentage (38.48%), indicating high variability for these traits. Low CV% was observed for grain length (3.23%), straw yield (3.26%), and grain width (5.17%), suggesting uniformity among genotypes for these traits.

### Analysis of variance and yield performance

**Fig 5:**
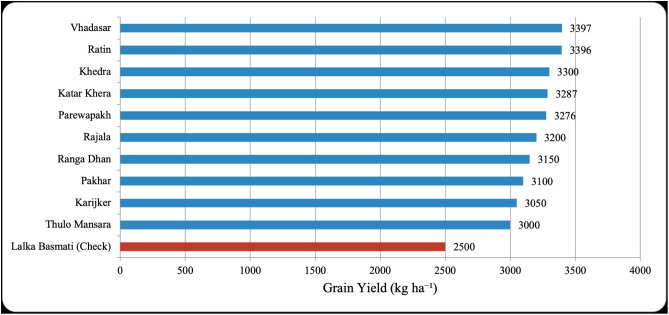
Top 10 high-yielding rice landraces compared to check variety (Lalka Basmati) at AFU, Rampur, Chitwan, 2018.

Analysis of variance revealed highly significant differences (p < 0.001) among genotypes for all 21 quantitative traits. Grain yield ranged from 433.00 to 3397.00 kg ha^−1^ with a grand mean of 1847.32 kg ha^−1^. The top-performing landraces were Vhadasar (3397 kg ha^−1^), Ratin (3396 kg ha^−1^), Khedra (3300 kg ha^−1^), Katar Khera (3287 kg ha^−1^), and Parewapakh (3276 kg ha^−1^), all exceeding the check variety Lalka Basmati (2500 kg ha^−1^) by 31–36% (Fig 5). Sixteen genotypes were found superior over the check variety for grain yield.

### Genetic variability parameters

In accordance with quantitative genetic theory, phenotypic coefficient of variation (PCV) exceeded genotypic coefficient of variation (GCV) for all traits, reflecting the contribution of environmental variance to total phenotypic variance. However, the magnitude of the difference between PCV and GCV, rather than the simple observation that PCV is higher than GCV, provides a relative indication of environmental influence on trait expression. A narrow difference between PCV and GCV was observed for grain length (GCV 21.78%, PCV 21.89%), straw yield (36.67%, 36.92%), grain width (14.19%, 14.53%), culm length (21.52%, 22.57%), and panicle length (15.82%, 16.60%), suggesting comparatively lower environmental influence and greater stability of these traits under the tested conditions. In contrast, a wider difference was observed for thousand-grain weight (GCV 15.76%, PCV 32.05%) and penultimate leaf length (GCV 4.33%, PCV 7.46%), indicating a relatively higher environmental contribution to the expression of these traits.

Broad-sense heritability was high (>60%) for most traits, with high genetic advance as percent of mean (>20%) for grain yield (H^2^b 81%, GAM 186%), filled grains per panicle (77%, 108%), harvest index (79%, 83%), panicle number per plant (75%, 71%), culm number per plant (76%, 68%) and productive tillers per m^2^ (75%, 63%). This shows that much of the variation in these traits is heritable and that they may respond well to phenotypic selection. However, because broad-sense heritability includes additive, dominance and epistatic variance, it cannot indicate the type of gene action; this would need further evidence, such as narrow-sense heritability or mating-design analysis. Thousand-grain weight was an exception, with low heritability (24%) and moderate GAM (15.96%), showing a smaller heritable share and a greater environmental effect (Table 3).

**Table 3:** Genetic variability parameters of quantitative traits in 64 rice genotypes at AFU, Rampur, Chitwan, 2018.

Traits	GCV (%)	PCV (%)	H²b (%)	GAM (%)
PLL	4.33	7.46	34	5.17
PLW	26.06	29.67	77	47.15
LLL	33.46	38.96	74	59.19
FLL	25.10	27.52	83	47.15
FLW	25.36	27.53	85	48.14
CL	21.52	22.57	91	42.25
CD	18.83	22.00	73	33.22
CNPP	37.79	43.36	76	67.84
PNPP	39.41	45.36	75	70.52
PL	15.82	16.60	91	31.07
FGPP	59.46	67.85	77	107.56
SSPP	81.16	87.80	85	154.42
SP	80.29	86.56	86	153.40
GL	21.78	21.89	99	44.63
GW	14.19	14.53	95	28.56
TGW	15.76	32.05	24	15.96
TTPM	23.46	27.79	71	40.80
PTPM	34.93	40.21	75	62.51
SY	36.67	36.92	99	75.04
GY	100.14	111.11	81	185.93
HI	45.07	50.64	79	82.65

PLL = Penultimate leaf length; PLW = Penultimate leaf width; LLL = Ligule length; FLL = Flag leaf length; FLW = Flag leaf width; CL = Culm length; CD = Culm diameter; CNPP = Culm number/plant; PNPP = Panicle number/plant; PL = Panicle length; FGPP = Filled grain/panicle; SSPP = Sterile spikelets/panicle; SP = Sterility %; GL = Grain length; GW = Grain width; TGW = 1000-grain weight; TTPM = Total tiller/m^2^; PTPM = Productive tiller/m^2^; SY = Straw yield; GY = Grain yield; HI = Harvest index; GCV = Genotypic coefficient of variation; PCV = Phenotypic coefficient of variation; H^2^b = Broad sense heritability; GAM = Genetic advance as percent of mean.

### Pearson’s correlation analysis

**Fig 6:**
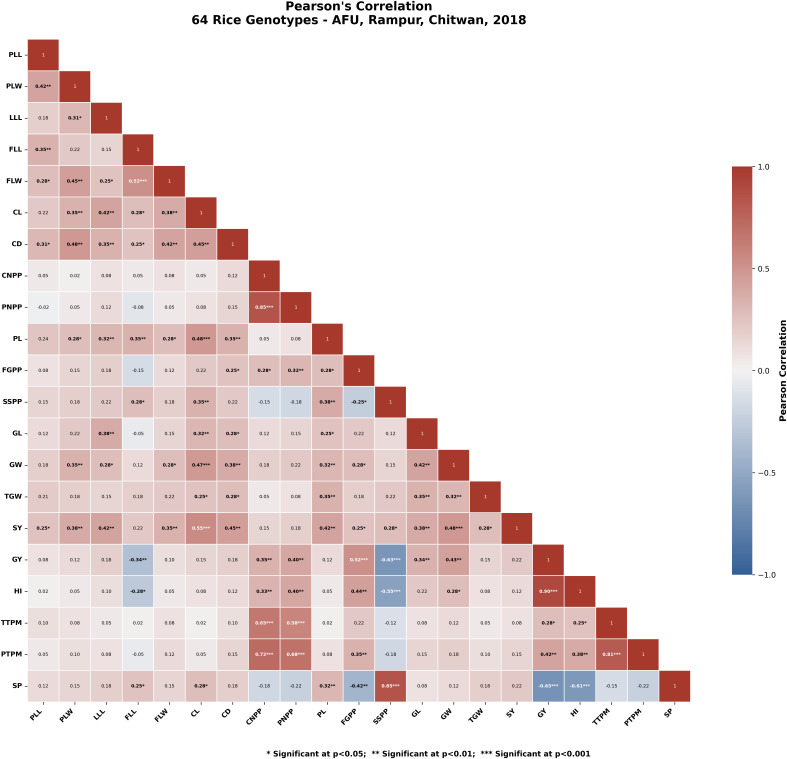
Pearson correlation matrix of 21 quantitative traits in 64 rice genotypes. Trait abbreviations are defined in Table 2. *, **, and *** indicate significance at p < 0.05, p < 0.01, and p < 0.001, respectively.

Pearson correlation coefficients among the 21 quantitative traits are presented in Fig 6. Grain yield was positively and significantly correlated with harvest index (r = 0.90***), filled grains per panicle (r = 0.52***), grain width (r = 0.43**), productive tillers per m^2^ (r = 0.42**), panicle number per plant (r = 0.40**), culm number per plant (r = 0.35**), grain length (r = 0.34**), and total tillers per m^2^ (r = 0.28*). It was negatively and significantly correlated with sterility percentage (r = –0.65***), sterile spikelets per panicle (r = –0.63***), and flag leaf length (r = –0.34**), and showed non-significant correlations with the remaining traits, including thousand-grain weight (r = 0.15) and straw yield (r = 0.22).

Harvest index followed a similar pattern, correlating positively with filled grains per panicle (r = 0.44**), panicle number per plant (r = 0.40**), culm number per plant (r = 0.33**), and grain width (r = 0.28*), and negatively with sterility percentage (r = –0.61***), sterile spikelets per panicle (r = –0.55***), and flag leaf length (r = –0.28*). The tillering traits were strongly intercorrelated: culm number with panicle number per plant (r = 0.85***), total with productive tillers per m^2^ (r = 0.81***), and productive tillers per m^2^ with culm number (r = 0.72***) and panicle number per plant (r = 0.68***).

Among the vegetative traits, flag leaf length correlated positively with flag leaf width (r = 0.52***) and penultimate leaf length (r = 0.35**); culm diameter with culm length (r = 0.45**), penultimate leaf width (r = 0.48**), and penultimate leaf length (r = 0.31*); and culm length with ligule length (r = 0.42**) and penultimate leaf width (r = 0.35**). Straw yield was positively correlated with culm length (r = 0.55***), grain width (r = 0.48***), culm diameter (r = 0.45**), ligule length (r = 0.42**), penultimate leaf width (r = 0.38**), and flag leaf width (r = 0.35**). Grain width was further associated with grain length (r = 0.42**) and thousand-grain weight (r = 0.32**), and sterility percentage correlated strongly with sterile spikelets per panicle (r = 0.85***) and negatively with filled grains per panicle (r = –0.42**).

### Principal component analysis

PCA helps to extract the independent variables that matter the most and gives direct measurement of total variance explained by few of the important components. Principal components and their respective proportion of the variation explained by eigenvalues and eigenvectors are presented (Table 4). Based on Kaiser’s criterion (eigenvalue >1), five principal components were extracted which cumulatively explained 73.40% of the total variation among 64 rice genotypes for 22 quantitative agro-morphological traits. The high amount of variation explained by PCA reflects the extent of phenotypic diversity present among the studied rice landraces.

**Table 4:** Eigenvalues, proportion of variance, and component loadings for the first five principal components derived from 22 quantitative traits in 64 rice genotypes.

Trait	PC1	PC2	PC3	PC4	PC5
Penultimate leaf length	−0.035	0.202	0.078	0.329	−0.110
Penultimate leaf width	−0.176	0.204	−0.409	0.110	−0.073
Ligule length	0.100	0.345	0.238	−0.039	0.008
Flag leaf length	−0.213	−0.096	−0.276	0.331	−0.190
Flag leaf width	−0.140	0.290	−0.368	0.198	−0.053
Culm diameter	−0.116	0.315	−0.043	0.318	−0.042
Culm length	−0.135	0.333	0.220	−0.093	−0.091
Culm number/plant	0.251	−0.157	0.148	0.303	−0.267
Panicle number/plant	0.291	−0.126	0.210	0.230	−0.248
Panicle length	−0.029	0.198	0.388	0.324	0.235
Filled grains/panicle	0.232	0.181	−0.010	0.086	0.411
Sterile grains/panicle	−0.296	−0.010	0.252	0.147	−0.040
Grain length	0.077	0.220	−0.114	−0.049	−0.415
Grain width	0.107	0.359	−0.031	0.005	−0.019
Moisture	−0.035	−0.150	0.055	0.502	0.210
Straw yield	0.116	0.352	0.200	−0.158	−0.148
Grain yield	0.332	0.133	−0.217	−0.003	−0.051
Harvest index	0.317	−0.009	−0.302	0.055	0.034
Total tillers/m²	0.294	−0.155	0.018	0.194	−0.126
Productive tillers/m²	0.359	0.027	−0.069	0.101	−0.011
Thousand grain weight	0.017	−0.046	0.159	−0.083	−0.522
Sterility	−0.335	−0.121	0.040	0.066	−0.213
Eigenvalue	6.026	4.726	2.206	1.689	1.494
Variance (%)	27.40	21.50	10.00	7.70	6.80
Cumulative (%)	27.40	48.90	58.90	66.60	73.40

Bold values indicate loadings ≥ |0.30|.

The first principal component (PC1) with eigenvalue 6.026 explained 27.40% of the total variation. Traits with high positive loadings on PC1 were productive tillers per m^2^ (0.359), grain yield (0.332), harvest index (0.317), total tillers per m^2^ (0.294), panicle number per plant (0.291), culm number per plant (0.251), and filled grains per panicle (0.232). Sterility (−0.335) and sterile grains per panicle (−0.296) exhibited high negative loadings on PC1. The second principal component (PC2) with eigenvalue 4.726 accounted for 21.50% of variance. High positive loadings were recorded for grain width (0.359), straw yield (0.352), ligule length (0.345), culm length (0.333), and culm diameter (0.315). The third component (PC3) with eigenvalue 2.206 explained 10.00% of variance, predominantly loaded with panicle length (0.388) and penultimate leaf width (−0.409). The fourth (PC4) and fifth (PC5) components contributed 7.70% and 6.80% of variance with eigenvalues 1.689 and 1.494, respectively. PC4 was mainly associated with grain moisture (0.502) and flag leaf length (0.331), while PC5 showed contrasting loadings for filled grains per panicle (0.411) and grain length (−0.415).

**Fig 7:**
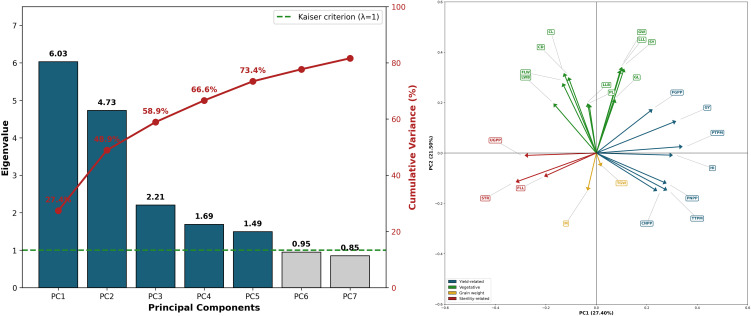
Principal component analysis: (a) Scree plot showing eigenvalues and cumulative variance explained by principal components; dashed line indicates Kaiser criterion (eigenvalue = 1). (b) PCA biplot showing relationship among 22 quantitative agro-morphological traits projected onto PC1 (27.40%) and PC2 (21.50%) in 64 rice genotypes.

Biplot of PCA analysis with first two principal components is presented (Fig 7). The first two components together explained 48.90% of the total variation. The graph clearly demarcated the distinct trait groups that dispersed along two principal component axes which emphasized on the extent of phenotypic variation among the rice landraces. Yield-related traits (grain yield, harvest index, productive tillers per m^2^, panicle number per plant, culm number per plant, filled grains per panicle) clustered in the positive PC1 direction, indicating their strong association with yield potential. Vegetative and morphological traits (straw yield, grain width, culm length, culm diameter, ligule length) grouped along the positive PC2 axis. Sterility and sterile grains per panicle were positioned in the negative PC1 quadrant, opposite to the yield-related traits, confirming the inverse relationship between sterility and grain yield. The positioning of traits in the biplot indicates that genotypes with high PC1 scores would exhibit superior yield performance with reduced sterility. Among 64 genotypes evaluated, landraces Vhadasar, Ratin, Khedra, and Katar Khera which were identified as high yielding in the present study are expected to possess high positive loadings for PC1. Our experimental results show that PCA analysis was able to identify the principal discriminatory characteristics viz., grain yield, harvest index, productive tillers per m^2^, panicle number per plant, filled grains per panicle, and sterility that contributed most to the differentiation among rice landraces.

### Cluster analysis

**Fig 8:**
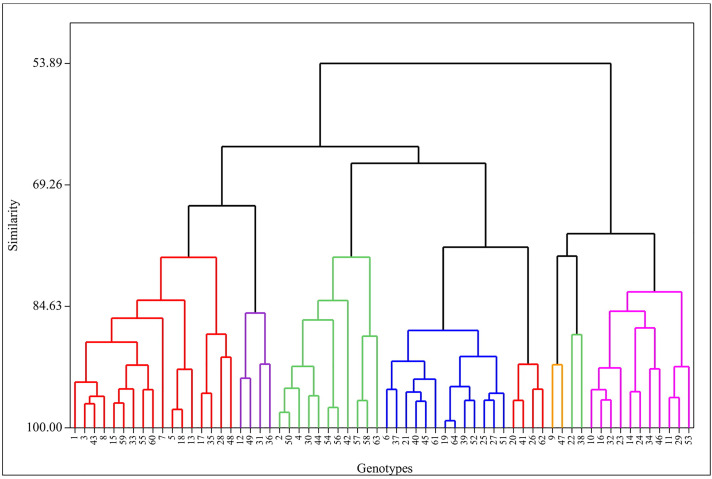
UPGMA (unweighted pair group method arithmetic average) clustering of 64 rice genotypes grown at Rampur, Chitwan, Nepal in 2018.

Cluster analysis was performed to group the 64 rice genotypes based on 22 quantitative agro-morphological traits using the UPGMA method. The dendrogram revealed eight distinct clusters at 53.89% similarity level (Fig 8). The cluster composition and distinguishing traits are summarized in Table 5.

**Table 5:** Relation of each cluster with distinct quantitative traits of rice landraces collected at Rampur, Chitwan, Nepal.

Cluster	Landraces	Distinct characters
**I**	Chhayang*, Rambela-An, Simtharo, Kalo Tulasi, Kusumkali, Sadharan Kalo Nuniya, Seto Dalle, Rato Anadi_J, Samalchauri, Dunminiya Seto, Rato Basmati, Malathe Ate, Kesarbachi, Madhumala, Sthaniya Sadhi, Ratagola-2 and Lalbachhi	Low grain yield, high sterility, low harvest index, tall culms
**II**	Karma, Marshi Dhan, Rato Tude Seto Basmati and Ujarka Jehariya	Tallest culms, high straw yield, lowest harvest index, low grain yield
**III**	Meethai, Najir, Rato Anadi-Na, Rato Andi, Thapachini, Ras Dhan, Rato Bachhi, Saauthyaariya, Rato Sthaniya, Rudrakshya and Thulo Mansara	High grain yield, high harvest index, high productive tillers per m^2^, low sterility, short culms
**IV**	Thapachini, Velasaro, Pakha, Laalchand, Tilaki*, Sete Dhan, Malbhog, Tilki, Lalka Basmati (Check), Rambela_J, Ramuni, Ratagola-1 and Pulingtar	Below-average grain yield, lowest straw yield, low thousand-grain weight
**V**	Nakhisaro, Ratanpuri, Rango and Sikichan	Lowest grain yield, highest sterility, longest flag leaves, most unfilled grains per panicle
**VI**	Kanakirawi and Komal Dhan	Lowest sterility, highest productive tillers per m^2^, high grain yield
**VII**	Pakhar and Vhadasar	Highest grain yield, high thousand-grain weight, high straw yield
**VIII**	Kanhar, Lalrenjer, Sakhar, Parewapakh, Khedra, Rajala, Siladhar, Katar Khera, Karijker, Ratin and Ranga Dhan	High grain yield, highest harvest index, highest filled grains per panicle, large grain width

Cluster I was the largest, with 17 genotypes (26.56%), characterized by low grain yield, high sterility (43.4%), low harvest index (17.2), and tall culms. Cluster II contained 4 genotypes (6.25%) with the tallest culms (127.6 cm) and high straw yield but the lowest harvest index (15.5). Cluster III comprised 11 genotypes (17.18%) with high grain yield (2307 kg ha^−1^), high harvest index (37.9), and high productive tillers per m^2^ (150.4), combined with low sterility and short culms. Cluster IV included 13 genotypes (20.31%), including the check variety, and showed below-average grain yield, the lowest straw yield, and low thousand-grain weight (23.4 g). Cluster V had 4 genotypes (6.25%) with the lowest grain yield (769 kg ha^−1^), the highest sterility (64.09%), the longest flag leaves (36.6 cm), and the most unfilled grains per panicle. Cluster VI and VII were the smallest clusters, with 2 genotypes each (3.12%); Cluster VI showed the lowest sterility (14.87%) and the highest productive tillers per m^2^ (180.34), while Cluster VII (Pakhar and Vhadasar) recorded the highest grain yield (3306 kg ha^−1^) together with high thousand-grain weight (25.9 g) and straw yield. Cluster VIII consisted of 11 genotypes (17.18%) with the highest harvest index (38.3) and the highest number of filled grains per panicle (100.7).

The wide distribution of genotypes across eight clusters indicates substantial phenotypic diversity among the studied rice landraces. Genotypes from Clusters VII, VIII, VI, and III exhibited superior yield performance and could serve as potential donors for yield improvement programs.

## Discussion

While rice genetic diversity has been extensively studied in South Asia, most evaluations have focused on gene bank accessions under conventional management. The present study uniquely characterized landraces sourced directly from community seed banks-the active custodians of on-farm diversity-and evaluated them under organic conditions without inorganic fertilizers. This approach captures genetic variation as maintained by farmers and assesses performance relevant to low-input systems that constitute over 70% of rice cultivation in Nepal’s hills and marginalized areas [25]. The extensive phenotypic diversity observed—polymorphism in 28 of 29 qualitative traits and significant variation (p < 0.001) for all 21 quantitative traits—confirms that community seed banks effectively conserve functional genetic diversity, supporting their role in decentralized conservation strategies.

A striking finding was that five landraces (Vhadasar, Ratin, Khedra, Katar Khera, Parewapakh) exceeded the improved check variety by 31–36% for grain yield under organic management. This superior performance likely reflects long-term farmer selection for productivity under low-input conditions, where traits such as nutrient use efficiency and root system architecture—often compromised in modern varieties bred under high-input regimes—remain intact [26]. The strong negative correlation between grain yield and sterility (r = −0.65***) underscores spikelet fertility as a primary yield determinant. Sterility directly reduces the number of filled grains per panicle, the sink capacity for photosynthate accumulation during grain filling. Genotypes with high sterility divert resources toward unfilled spikelets, resulting in lower harvest index and grain yield—a pattern consistently observed across rice germplasm [27,28].

Another notable result was the negative correlation of flag leaf length with both grain yield (r = –0.34**) and harvest index (r = –0.28*). This appears to differ from the general understanding that the flag leaf, as the main source of assimilates during grain filling, contributes positively to yield, and from earlier studies reporting a positive association between flag leaf size and yield in rice [29]. Two factors may explain this difference. First, flag leaf length is not equivalent to flag leaf width or area, which are more directly related to photosynthetic capacity and yield; flag leaf width, in particular, has been associated with higher yield, whereas flag leaf length frequently shows a weak or non-significant relationship with it [30]. In the present study, flag leaf length was correlated mainly with flag leaf width (r = 0.52***) and penultimate leaf length (r = 0.35**)—that is, with overall leaf size—rather than with any yield component, suggesting that it behaved primarily as a vegetative trait. Second, its negative association with harvest index suggests that genotypes with longer flag leaves tended to be taller, more vegetative traditional landraces that allocated comparatively less biomass to grain. A similar negative association between flag leaf length and grain yield has been reported in Nepalese rice landraces [20], indicating that this pattern is not unique to the present material but may be characteristic of traditional Nepalese landraces, in which long flag leaves are often accompanied by tall, low-harvest-index plant types. Taken together, these results suggest that flag leaf width or area, together with harvest index, may be more reliable selection criteria than flag leaf length for improving yield in this germplasm. Consistent with this, harvest index showed the strongest positive association with grain yield (r = 0.90***), indicating that yield in this germplasm was driven more by efficient partitioning of biomass to grain than by total vegetative growth, in line with the non-significant straw-yield correlation (r = 0.22).

The high broad-sense heritability and high genetic advance as percent of mean recorded for grain yield, filled grains per panicle, and productive tillers per m^2^ indicate that a substantial and heritable proportion of the phenotypic variation in these traits is attributable to genotypic differences, identifying them as promising targets for phenotypic selection. Comparable estimates of high heritability coupled with high genetic advance for grains and filled grains per panicle, productive tillers, harvest index, and grain yield have been widely reported in rice [31,32], and similar patterns have been documented in Nepalese rice germplasm and landraces evaluated in the mid-hills and Terai [33,34]. The wide phenotypic range characteristic of landrace collections, also reported in earlier Nepalese germplasm [33], is consistent with the large coefficients of variation and high genetic-advance values obtained in the present study. Such combinations of high heritability and high genetic advance are frequently interpreted as evidence of additive gene action. However, broad-sense heritability includes additive, dominance, and epistatic variance, and therefore cannot, by itself, establish the underlying mode of gene action. Demonstrating additive control, or the effectiveness of selection in early generations, would require additional evidence such as narrow-sense heritability estimates or appropriate genetic and mating-design analyses. These parameters are therefore interpreted here as indicating substantial genetic variability and a favourable potential for response to phenotypic selection, rather than as evidence of a specific mode of gene action.

The narrow differences between PCV and GCV observed for most traits, reflecting a limited environmental contribution, are in agreement with earlier rice studies in which closely corresponding phenotypic and genotypic coefficients of variation indicated predominantly genetic control [34]. Thousand-grain weight, in contrast, showed low broad-sense heritability (24%) with only moderate genetic advance (GAM 15.96%), indicating that a comparatively small proportion of its variation was genetic, and that environmental or residual variation contributed substantially to its expression under the present conditions. Because the present study was conducted in a single environment, the genotype-by-environment component of variation could not be estimated; the low heritability of this trait should not, therefore, be attributed to genotype-by-environment interaction or to non-additive gene action but interpreted more conservatively as a small genetic variance relative to the total phenotypic variance. Characterizing the environmental sensitivity of thousand-grain weight would require evaluation across multiple environments or seasons.

Multivariate analyses revealed interpretable genetic structure. PC1 (27.4% variance) represented a yield-sterility axis, with high-yielding, low-sterility genotypes at one extreme and poor performers at the other. This dimension captures the fundamental trade-off between reproductive success (filled grains) and failure (sterile spikelets). PC2 (21.5% variance) reflected vegetative vigor and grain size, traits often associated with traditional landraces adapted to compete with weeds and produce bold grains preferred in local markets. The eight UPGMA clusters further partitioned this variation: Cluster IV combined favorable alleles for yield components (highest productive tillers, filled grains, lowest sterility), explaining why 13 genotypes in this group collectively outperformed others. Cluster VIII accumulated large-grain genotypes including top performers Khedra and Ratin, suggesting grain size contributes to yield through increased individual grain weight compensating for moderate tiller numbers. Such complementary trait architectures across clusters offer opportunities for transgressive segregation through inter-cluster hybridization [29].

These findings have direct implications for multiple stakeholders. For farmers, landraces Vhadasar, Ratin, Khedra, and Katar Khera represent immediately deployable varieties for organic and low-input systems without requiring external seed purchase or changed practices. For breeders, these landraces serve as donor parents for introgressing yield-enhancing alleles into elite backgrounds; the identified selection criteria (filled grains per panicle, productive tillers, low sterility) provide a phenotyping framework for progeny evaluation. For policy makers and conservationists, the genetic structure across eight clusters—with smaller clusters potentially harboring unique adaptive variants—supports targeted conservation. Community seed banks in Jhapa, Bara, Nawalparasi, and Dang districts, which supplied these landraces, merit continued support as critical nodes in Nepal’s seed system maintaining diversity that formal gene banks may not capture.

This study characterized landraces at a single location under organic management; multi-environment evaluation would strengthen genotype recommendations and quantify stability. Molecular characterization would complement phenotypic assessment by resolving genetic relationships independent of environment and enabling marker-assisted selection for complex traits. Nevertheless, the phenotypic diversity and superior performers identified here provide an empirical foundation for both immediate utilization and longer-term genetic improvement of rice in Nepal.

## Conclusion

This study revealed considerable agro-morphological diversity among Nepalese rice landraces maintained in community seed banks. Landraces Vhadasar, Ratin, Khedra, Katar Khera, and Parewapakh demonstrated superior yield potential, outperforming the improved check variety by 31–36% under organic conditions. High heritability combined with high genetic advance for yield-related traits indicated that simple selection would be effective for genetic improvement. The negative association between sterility and grain yield highlighted spikelet fertility as a priority trait for selection. Cluster analysis identified genetically diverse groups suitable for hybridization programs. These findings underscore the importance of community seed banks in conserving valuable genetic resources and provide a foundation for developing high-yielding, climate-resilient rice varieties for sustainable agriculture in Nepal and similar agro-ecosystems.

## Supporting information

S1 Fig Grain diversity among 64 Nepalese rice landraces showing variation in grain color, shape, and size. Each pile represents seeds from individual landraces with their local names labeled.
(TIF)

S2 Fig Panicle diversity among 64 Nepalese rice landraces displaying variation in panicle length, branching pattern, grain density, and overall architecture. (TIF)

S1 Table List of 29 qualitative traits, with descriptor states, growth stage, and method of observation. (DOCX)

S2 Table List of 21 quantitative traits, with units, growth stage, and measurement method. (DOCX)

S3 File Underlying dataset for all 64 rice genotypes. (XLSX)
