# Supplementary figures and images for: Characterization and evaluation of Nepalese rice landraces using agro-morphological traits
Source: PLoS One. 2026 Aug 3;21(8):e0348162. doi: 10.1371/journal.pone.0348162 (PMC13432108; doi:10.1371/journal.pone.0348162)

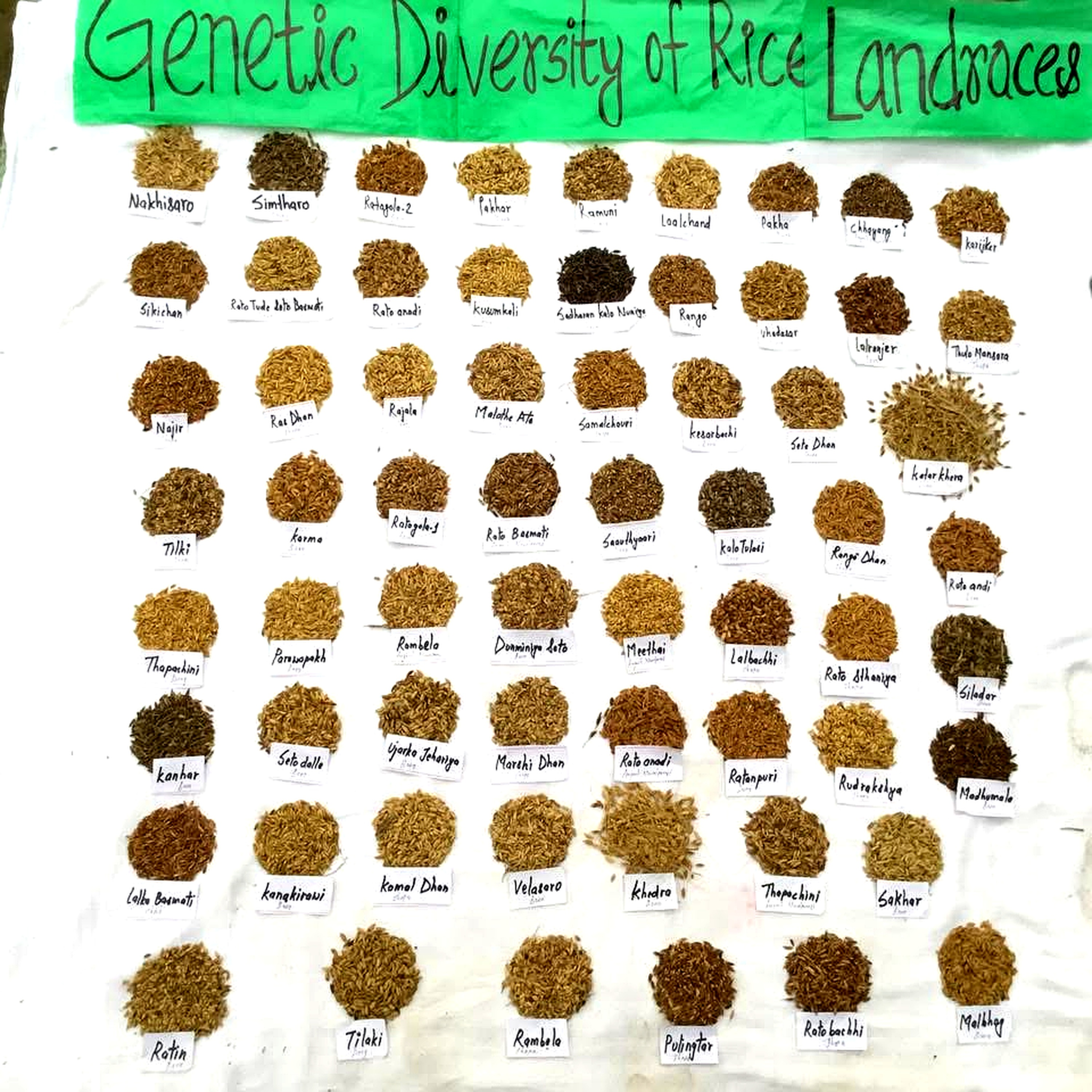

Supplement: S1 Fig — Each pile represents seeds from individual landraces with their local names labeled. (TIF) [file pone.0348162.s001.tif]

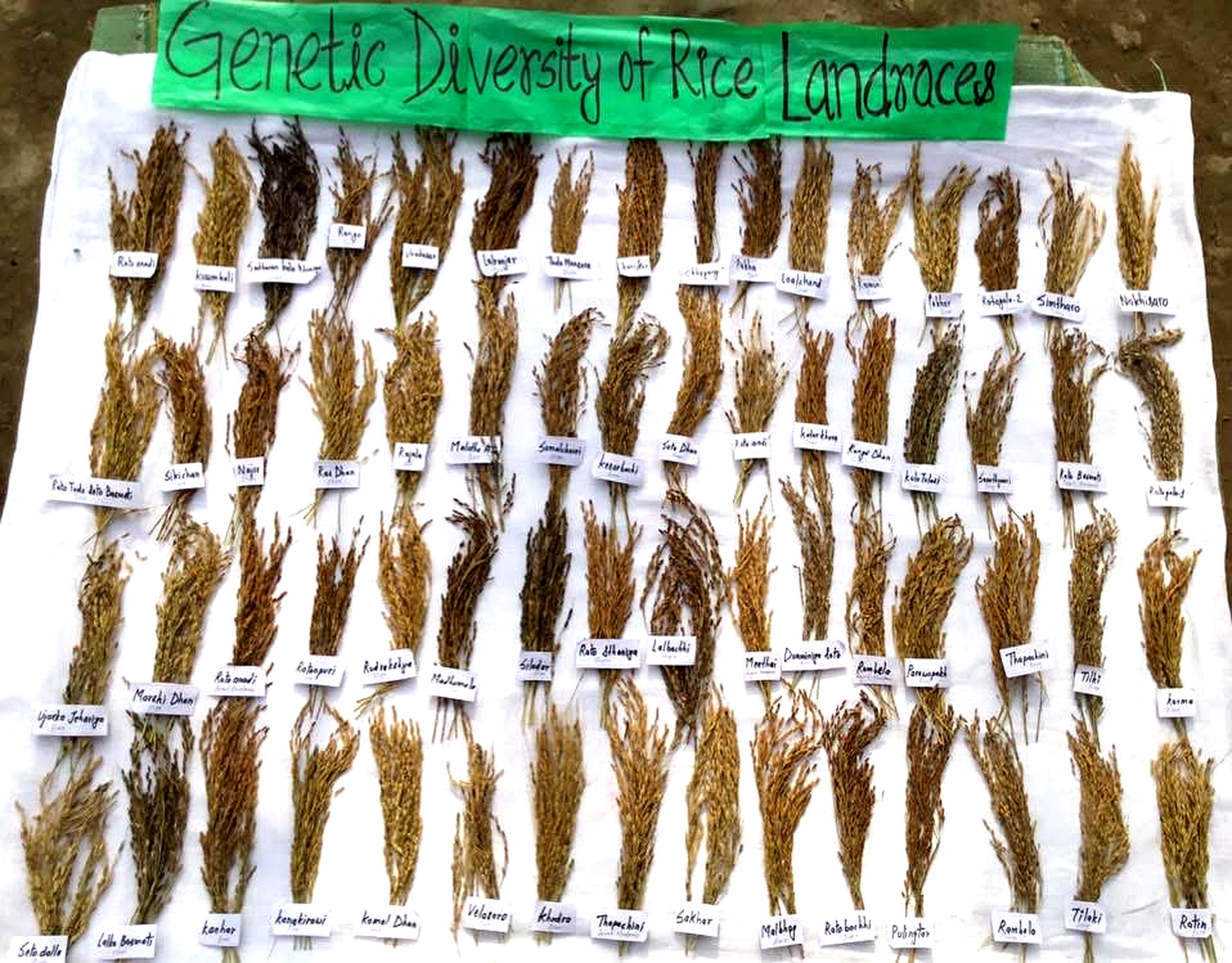

Supplement: S2 Fig — (TIF) [file pone.0348162.s002.tif]
