# Supplementary material for: Characterization and evaluation of Nepalese rice landraces using agro-morphological traits
Source: PLoS One. 2026 Aug 3;21(8):e0348162. doi: 10.1371/journal.pone.0348162 (PMC13432108; doi:10.1371/journal.pone.0348162)
Supplement: S1 Table — (DOCX) [file pone.0348162.s003.docx]

**S1 Table. Qualitative agro-morphological traits recorded in 64 rice genotypes, with descriptor states, crop stage, and method of observation.**

| **SN** | **Qualitative trait** | **Descriptor states** | **Crop stage** | **Method** |
| --- | --- | --- | --- | --- |
| 1 | Basal leaf sheath colour | Green; green with purple lines; light purple; purple | Late vegetative | Visual |
| 2 | Leaf blade: intensity of green colour | Light; medium; dark | Late vegetative | Visual |
| 3 | Leaf blade: pubescence | Glabrous; intermediate; pubescent | Late vegetative | Visual + tactile |
| 4 | Leaf blade: anthocyanin coloration | Absent; present | Late vegetative | Visual |
| 5 | Leaf sheath: anthocyanin coloration | Absent; weak; medium; strong | Late vegetative | Visual |
| 6 | Leaf blade attitude | Erect; horizontal; drooping | Late vegetative (pre-heading) | Visual |
| 7 | Flag leaf: attitude of blade (early) | Erect; semi-erect; horizontal; descending | Anthesis | Visual |
| 8 | Flag leaf: attitude of blade (late) | Erect; semi-erect; horizontal; descending | Maturity | Visual |
| 9 | Auricle colour | Absent; whitish; yellowish-green; purple; light purple; purple lines | Late vegetative | Visual |
| 10 | Collar colour | Absent; green; light green; purple; purple lines | Late vegetative | Visual |
| 11 | Ligule colour | Absent; whitish; yellowish-green; purple; light purple; purple lines | Late vegetative | Visual |
| 12 | Ligule shape | Absent; truncate; acute to acuminate; 2-cleft | Late vegetative | Visual |
| 13 | Culm: kneeing ability | Absent; present | After flowering | Visual |
| 14 | Culm: habit (angle) | Erect; semi-erect; open; spreading; procumbent | After flowering | Visual |
| 15 | Culm: anthocyanin coloration of nodes | Absent; purple; light purple; purple lines | Flowering–near maturity | Visual |
| 16 | Culm: underlying node colour | Light gold; green | Flowering–near maturity | Visual |
| 17 | Culm: internode anthocyanin coloration | Absent; purple; purple lines | Near maturity | Visual |
| 18 | Culm: underlying internode coloration | Light gold; green | Near maturity | Visual |
| 19 | Culm: lodging resistance | Very weak; weak; intermediate; strong; very strong | Maturity | Visual |
| 20 | Awns: colour | Absent; whitish; straw; gold; brown; light green; red; purple; black | After anthesis | Visual |
| 21 | Awns: distribution | Absent; tip only; upper quarter; upper half; upper three-quarters; whole length | Flowering–maturity | Visual |
| 22 | Apiculus colour | White; straw; brown; green; red; red apex; purple; purple apex; black | After anthesis to hard dough | Visual |
| 23 | Lemma and palea colour | White; gold furrows; brown (tawny); brown spots/furrows on green; green; yellowish-green; purple; purple shade/spots/furrows; black | After anthesis to hard dough | Visual |
| 24 | Lemma: anthocyanin coloration below apiculus | Absent; very weak; weak; medium; strong | After anthesis to hard dough | Visual |
| 25 | Stigma colour | White; light green; yellow; light purple; purple | Anthesis | Visual, hand lens |
| 26 | Panicle: exsertion | Enclosed; partly exserted; just exserted; moderately well exserted; well exserted | Near maturity | Visual |
| 27 | Panicle: attitude of main axis | Upright; semi-upright; slightly drooping; strongly drooping | Near maturity | Visual |
| 28 | Panicle: attitude of branches | Erect (compact); semi-erect; spreading (open); horizontal; drooping | Near maturity | Visual |
| 29 | Panicle: secondary branching | Absent; sparse; dense; clustered | Near maturity | Visual |

Traits, descriptor states and recording stages follow Descriptors for wild and cultivated rice (Oryza spp.), Bioversity International, IRRI and WARDA (2007). Qualitative traits were recorded by visual assessment (visual-grade scoring); quantitative traits by measurement of several individual samples (MS) or a single measurement of a group (MG).
