# Supplementary material for: Characterization and evaluation of Nepalese rice landraces using agro-morphological traits
Source: PLoS One. 2026 Aug 3;21(8):e0348162. doi: 10.1371/journal.pone.0348162 (PMC13432108; doi:10.1371/journal.pone.0348162)
Supplement: S2 Table — (DOCX) [file pone.0348162.s004.docx]

**S2 Table. Quantitative agro-morphological traits recorded in 64 rice genotypes, with units, crop stage, and method of measurement.**

| **SN** | **Quantitative trait (abbr.)** | **Unit** | **Crop stage** | **Method of measurement** |
| --- | --- | --- | --- | --- |
| 1 | Penultimate leaf length (PLL) | cm | Early reproductive | MS – ligule to blade tip; mean of 5 plants, graduated scale |
| 2 | Penultimate leaf width (PLW) | cm | Early reproductive | MS – widest point of blade; mean of 5 plants, graduated scale |
| 3 | Ligule length (LLL) | cm | After anthesis | MS – base of collar to ligule tip; mean of 5 plants, graduated scale |
| 4 | Flag leaf length (FLL) | cm | 7 days after anthesis | MS – ligule to blade tip; mean of 5 plants, graduated scale |
| 5 | Flag leaf width (FLW) | cm | 7 days after anthesis | MS – widest point of blade; mean of 5 plants, graduated scale |
| 6 | Culm length (CL) | cm | After flowering to maturity | MS – ground level to panicle base; mean of 5 main culms, graduated scale |
| 7 | Culm diameter (CD) | mm | Flowering / late reproductive | MS – basal internode outer diameter; mean of 3 plants, vernier caliper |
| 8 | Culm number per plant (CNPP) | count | After anthesis to near maturity | MS – total grain-bearing and non-bearing tillers; mean of 5 plants |
| 9 | Panicle number per plant (PNPP) | count | Early ripening | MS – panicles per plant; mean of 5 plants |
| 10 | Panicle length (PL) | cm | After harvest | MS – base to tip of main axis; mean of 5 plants, graduated scale |
| 11 | Filled grains per panicle (FGPP) | count | After harvest | MS – filled grains on panicles of 5 plants, averaged |
| 12 | Sterile spikelets per panicle (SSPP) | count | After harvest | MS – sterile spikelets on panicles of 5 plants, averaged |
| 13 | Sterility (SP) | % | After harvest | Derived – sterile spikelets as % of total spikelets on 5 panicles |
| 14 | Grain length (GL) | mm | After harvest | MS – mean of 10 grains, vernier caliper |
| 15 | Grain width (GW) | mm | After harvest | MS – mean of 10 grains, micrometer screw gauge |
| 16 | Thousand-grain weight (TGW) | g | After harvest | MG – 1000 whole grains dried to 13% moisture, precision balance |
| 17 | Total tillers per m² (TTPM) | count | Early ripening | MG – all tillers counted in a 1 m² net area |
| 18 | Productive tillers per m² (PTPM) | count | Early ripening | MG – panicle-bearing tillers counted in a 1 m² net area |
| 19 | Straw yield (SY) | kg ha⁻¹ | At harvest | MG – whole-plot straw weight (sun-dried), converted to kg ha⁻¹ |
| 20 | Grain yield (GY) | kg ha⁻¹ | At harvest | MG – whole-plot grain weight; moisture-corrected to 13% and converted to kg ha⁻¹ |
| 21 | Harvest index (HI) | % | At harvest | Derived – grain yield / biological yield (grain + straw) × 100 |

Leaf descriptors (sheath, blade, ligule, auricle, collar) were recorded on the penultimate leaf. Unless otherwise stated, quantitative traits were measured on five randomly selected representative plants per plot and averaged; grain and straw yields were recorded on a whole-plot basis. Thousand-grain weight was determined from 1000 well-developed grains dried to 13% moisture content*.*
